# Detection of emerging neurodegeneration using Bayesian linear mixed-effect modeling

**DOI:** 10.1016/j.nicl.2022.103144

**Published:** 2022-08-06

**Authors:** Yann Cobigo, Matthew S. Goh, Amy Wolf, Adam M. Staffaroni, John Kornak, Bruce L. Miller, Gil D. Rabinovici, William W. Seeley, Salvatore Spina, Adam L. Boxer, Bradley F. Boeve, Lei Wang, Ricardo Allegri, Marty Farlow, Hiroshi Mori, Richard J. Perrin, Joel Kramer, Howard J. Rosen, for the Alzheimer’s Disease Neuroimaging Initiative (ADNI) and the Dominantly Inherited Alzheimer’s Network (DIAN)

**Affiliations:** a University of California, San Francisco, Department of Neurology, Memory and Aging Center, United States; b University of California, San Francisco, Department of Epidemiology and Biostatistics, United States; c Mayo Clinic, Rochester, Department of Neurology, United States; d Northwestern University Feinberg School of Medicine, Department of Psychiatry and Behavioral Sciences and Department Radiology, United States; e FLENI Institute of Neurological Research (Fundacion para la Lucha contra las Enfermedades Neurologicas de la Infancia), Argentina; f Indiana University, United States; g Osaka City University Medical School, Department of Neurosciences, Japan; h Washington University School of Medicine, United States

**Keywords:** Bayesian linear mixed-effect, Bayesian prediction, Alzheimer’s Disease, Frontotemporal Lobar Degeneration

## Abstract

Early detection of neurodegeneration, and prediction of when neurodegenerative diseases will lead to symptoms, are critical for developing and initiating disease modifying treatments for these disorders. While each neurodegenerative disease has a typical pattern of early changes in the brain, these disorders are heterogeneous, and early manifestations can vary greatly across people. Methods for detecting emerging neurodegeneration in any part of the brain are therefore needed. Prior publications have described the use of Bayesian linear mixed-effects (BLME) modeling for characterizing the trajectory of change across the brain in healthy controls and patients with neurodegenerative disease. Here, we use an extension of such a model to detect emerging neurodegeneration in cognitively healthy individuals at risk for dementia. We use BLME to quantify individualized rates of volume loss across the cerebral cortex from the first two MRIs in each person and then extend the BLME model to predict future values for each voxel. We then compare observed values at subsequent time points with the values that were expected from the initial rates of change and identify voxels that are lower than the expected values, indicating accelerated volume loss and neurodegeneration. We apply the model to longitudinal imaging data from cognitively normal participants in the Alzheimer’s Disease Neuroimaging Initiative (ADNI), some of whom subsequently developed dementia, and two cognitively normal cases who developed pathology-proven frontotemporal lobar degeneration (FTLD). These analyses identified regions of accelerated volume loss prior to or accompanying the earliest symptoms, and expanding across the brain over time, in all cases. The changes were detected in regions that are typical for the likely diseases affecting each patient, including medial temporal regions in patients at risk for Alzheimer’s disease, and insular, frontal, and/or anterior/inferior temporal regions in patients with likely or proven FTLD. In the cases where detailed histories were available, the first regions identified were consistent with early symptoms. Furthermore, survival analysis in the ADNI cases demonstrated that the rate of spread of accelerated volume loss across the brain was a statistically significant predictor of time to conversion to dementia. This method for detection of neurodegeneration is a potentially promising approach for identifying early changes due to a variety of diseases, without prior assumptions about what regions are most likely to be affected first in an individual.

## Highlights

• Sensitive method for early detection of neurodegenerative cluster trajectories.
• Individual Bayesian prediction formalism based on group linear mixed-effect modeling.
• Survival analysis showed that the rate of spread is a predictor of time to dementia.

## 1 Introduction

Neurodegenerative disorders are increasingly common causes of disability and death in the population Association (2019). Extensive efforts to develop new treatments for these disorders are underway, and such treatments may be most effective if they are initiated early in the course of disease Sperling et al., 2014, Boxer et al., 2020. The biological processes underlying neurodegenerative diseases begin many years before the development of symptoms Jack et al., 2013, Rosen et al., 2020b, Weiner et al., 2017. Intervention during the asymptomatic phase offers the possibility of delaying or preventing onset of symptoms Sperling et al., 2014, Boxer et al., 2020. Implementation of such a preventative approach, either in research or clinical care, requires measurements to detect that the neurodegenerative process has begun and symptoms are on the horizon. In the case where a drug has adverse effects, a reasonable strategy might be to delay treatment until near the end of the presymptomatic phase. Commonly cited models, supported by empirical data Staffaroni et al., 2020b, McDade et al., 2018 stipulate that many biological markers (biomarkers) of neurodegenerative disease evolve to become more abnormal over time, with slow rates of change when people are healthy and/or in the early phase of disease, and acceleration preceding or accompanying symptom onset Jack et al., 2013, Araque Caballero et al., 2015. Identifying when biomarkers enter this phase of accelerated change may be particularly valuable for predicting oncoming symptoms.

Among the growing list of biomarkers, regional brain volume (rBV) measured from T1-weighted MRI is an attractive measure for detecting neurodegeneration Jack et al. (2016) and oncoming symptoms. A vast body of literature has demonstrated that rBV is correlated with a variety of symptoms in neurodegenerative disease Rosen et al., 2005, Kramer et al., 2005, Rosen and Levenson, 2009, Krueger et al., 2011, and that both the degree of accumulated brain volume loss Dickerson et al., 2011, McEvoy et al., 2011, Staffaroni et al., 2020a, Paulsen et al., 2014 and rate of brain volume loss Dodge et al., 2014, Chen et al., 2020, Chen et al., 2019, are valuable for predicting decline in cognition in patients at increased risk for neurodegeneration (due to aging, genetics, or other factors). Many of these studies have optimized the use of rBV for prediction by focusing on à priori or empirically identified regions based on the tendency for specific diseases to affect particular brain regions. For example, hippocampal volume (HV), which is commonly reduced in Alzheimer’s disease (AD), has been highlighted as a useful measure for predicting clinical decline in those at high risk for AD Hill et al., 2014, Yu et al., 2014. Many studies have also highlighted the utility of other brain regions in AD McEvoy et al. (2011) and other neurodegenerative disorders Staffaroni et al. (2019), but most have assumed that a given set of regions would be utilized to detect oncoming symptoms in all patients with that disease. Yet, it is well established that the nature of early symptoms in neurodegenerative disorders varies across people. In AD, for example, a substantial subset of patients, particularly those with early age of onset, first develop language, dysexecutive, behavioral, or visuospatial symptoms reflecting early frontal and parietal changes, rather than memory loss indicative of medial temporal/hippocampal involvement Ossenkoppele et al. (2015). In frontotemporal lobar degeneration (FTLD), the same pathology can present with behavioral, dysexecutive, or movement symptoms, or with multiple forms of language dysfunction Olney et al. (2017). Given that the earliest symptom for a given patient cannot be predicted, there is a need for individualized measures that detect emergence of neurodegeneration in any part of the brain without à priori assumptions about where the disease will begin.

Although early detection might be improved with individualized approaches, exclusive focus on the earliest region of involvement may limit the prediction of symptoms. Many biological models of neurodegeneration propose the spread of toxic proteins across functionally specialized brain networks Seeley et al., 2009, Raj et al., 2012, Jones et al., 2017. Indeed, studies have indicated that brain network architecture predicts spread of atrophy from the earliest site of involvement to other regions of the brain Brown et al. (2019). While limited changes in any part of the brain may account for early symptoms, impairments with significant impact on daily function (*i.e.* dementia) result from progressive involvement of more brain regions over time McEvoy et al., 2011, Staffaroni et al., 2020a. An approach that is sensitive to the earliest changes but also quantifies the spread of disease to additional brain regions might therefore provide the best method for predicting symptom onset, in addition to predicting which symptoms will occur first.

A prior publication by Ziegler and colleagues introduced a framework to quantify longitudinal trajectories of brain volume using hierarchical linear mixed-effects models with Bayesian inference Ziegler et al. (2015). The approach was applied to group-level data to create probabilistic maps of the mean and variance in rates, and acceleration, of volume loss at every location in the brain. Here, we describe an extension of the method that uses the Bayesian framework to estimate the rate of gray matter loss at every region in the brain from MRIs in individuals who are cognitively normal, and to identify regions in subsequent MRIs where gray matter loss exceeds expectations in those individuals (based on the estimates from the initial MRIs), indicating accelerated gray matter loss and emerging neurodegeneration. While the chief purpose of this paper is to describe the method, we also sought to illustrate its use in a relevant population. Therefore, after describing the method, we report on the application of the method in a group of research participants who were followed longitudinally, some of whom went on to develop dementia, to test whether this approach can be used to predict the development of dementia without à priori assumptions about where in the brain atrophy is likely to occur. For this purpose, we used participants at risk for AD who enrolled in the Alzheimer’s disease neuroimaging initiative (ADNI). Although there are many methods for predicting dementia in people at risk for AD, ADNI is an ideal resource for testing our model because of the large number of participants with prolonged followup. We then proceed to illustrate how this approach can be useful in other dementias where prediction methods are less well-developed, using case examples.

## 2 Materials and Methods

### 2.1 Overview

Our approach uses Bayesian modeling to estimate and predict the trajectory of changes in gray matter content from T1-weighted MRIs at every volume element (voxel) in the gray matter in individual participants of interest. In this study, participants of interest are individuals at risk for neurodegeneration who are cognitively normal at the time they are initially studied. Estimation of the rate of gray matter loss in each individual is accomplished by including their first two longitudinal MRIs in a linear mixed-effects (LME) model, along with longitudinal MRIs from a group of demographically matched participants (in this case, a group of cognitively normal individuals whose age range includes the age of the individual of interest), to estimate the relationship between gray matter content at each voxel and time for the group as a whole and for each individual.

Estimating the trajectory of changes in regional volumes for a single individual from an LME model, instead of using only the observed data from this individual, provides an improved estimate for predicting future values of brain volume because the estimated group effects, and uncertainties, are used to provide additional group level information to the individual estimates. “Borrowing strength” in this way lessens the impact of random factors that could affect an individual observation, such as movement and other artifacts that negatively influence image quality and image summary estimation. In this study, we use the Bayesian framework to estimate model parameters. This framework uses à priori beliefs to define prior probability distributions for the parameters of interest, and the prior probability distribution is updated using the data to provide posterior probability distributions. In contrast to classical statistical approaches, the Bayesian framework quantifies uncertainty by estimating the model fit for all possible values of all parameters, rather than optimizing them to produce a single best estimate vector (*e.g.* Maximum Likelihood). Therefore, information about the parameters is carried forward in the form of a probability density distribution that captures all the knowledge, including uncertainty, about the parameter. Given that our goal is to identify regions that are lower in volume than expected based on the initial observations from each individual, these probability density functions are ideal for our purposes because they can be used to create probability estimates for each potential value at future observations, as described below.Because the parameters are constrained or regularized by the priors, this approach is also one method to avoid overfitting Ghahramani (2001).

Once the model posterior parameters are established from the initial observations, the Bayesian framework is used to establish the predictive probability density functions for future observations based on the previously modeled rate of change Gelman et al., 2013, Beal, 2003. In order to evaluate the state of gray matter content at each time point for each person, we can compare the observed gray matter content at each voxel from subsequent observations in that person that were not included as data in the Bayesian LME (BLME) model with the predictive probability density functions for gray matter content at those time points. Regions where the probability densities for the calculated values are at the extremes of the probability distribution represent locations where the gray matter content is far from values that would have been most likely if gray matter loss had proceeded at the originally estimated rate.

**Fig. 1:**
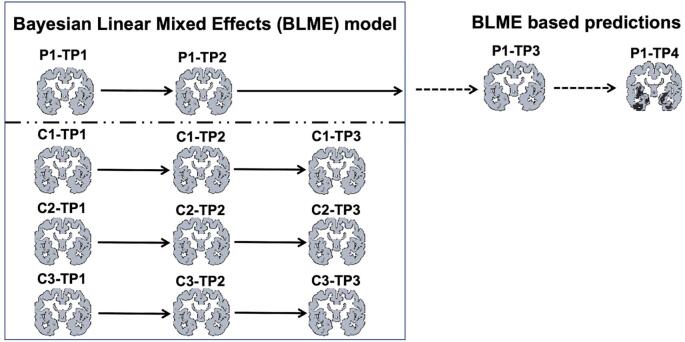
Schematic depicting the method for identifying voxels affected by neurodegeneration using longitudinal imaging. In this depiction, the first two time points (TP1, TP2) from a participant of interest (P1) are introduced into the BLME model along with three time points from three control participants (C1, C2, C3). The BLME parameters are used to estimate the rate of volume loss at every voxel for every individual, and these estimates are used to predict the volumes for each voxel at time points 3 and 4 for P1. The predicted values are compared with the observed values, and observed values that are far lower than the predicted values (darker regions in TP4) are assumed to be undergoing neurodegeneration.

If there are a number of individuals for whom one wishes to predict future observations, the process of estimating and predicting probability distributions for observations of every voxel in the brain is done separately for each individual of interest by including each one in a BLME model with a group of matched cases. If the group of matched cases is appropriately chosen to span the range of age and other relevant variables for all of the individuals of interest, the same group of matched cases can be used for all individuals of interest. This produces individualized estimates of change across the gray matter voxels from each subject’s initial images, and maps of voxels with unexpectedly large amounts of gray matter loss at later time points for each person, considering the prior estimate of rate of change in that individual. The process is illustrated for one individual in the schematic in Fig. 1. As a form of linear mixed-effects modeling, BLME supports inhomogeneous time series with different numbers of time points and irregular time spacing between acquisitions.

In the following sections, we briefly review the BLME model using the same notations and formulas as those in the original description, and refer the reader to prior publications for a more thorough description Ziegler et al. (2015). We then describe the extension of the model to extract the Bayesian prediction models for single subjects. The BLME and prediction algorithms were implemented in C++ using the Insight Toolkit (ITK, version 4.9) library Johnson et al., 2015a, Johnson et al., 2015b, and linear algebra components were developed using Eigen3 C++ headers Guennebaud and Jacob (2010). The source code will be freely available on github.

### 2.2 BLME and Posterior Distribution Calculation

In order to estimate the trajectory of volume change at each voxel for each individual from their first two images, we incorporated their images into Bayesian linear mixed-effects models using the approach described by Ziegler et al. (2015). Here, we provide only the information necessary for understanding the calculations used to implement our extension of the model for prediction of future voxel volumes that is described in the next section. Full details regarding the calculations necessary for implementing the model to estimate initial trajectories are provided in Section 7.1. For each individual, the trajectory y is fitted as a straight line with a random intercept and slope, Eq. (7). We modeled a hierarchical structure with two levels. The first level is defined by the trajectory model with the design matrix $X^{\left(1\right)}$, representing the temporal elements from each subject (constant, first, second, …, orders of the temporal polynomial). The second level design matrix $X^{\left(2\right)}$ represents the contribution of subject’s covariates, *e.g.* total intracranial volume (TIV).

$$y=\left[\begin{array}{cc} X^{\left(1\right)} & X^{\left(1\right)}X^{\left(2\right)} \end{array}\right]\left[\begin{array}{c} {∊}^{\left(2\right)}\\ \theta^{\left(2\right)} \end{array}\right]+{∊}^{\left(1\right)}\text{.}$$

At each level, the noise is considered as a centered Gaussian distribution: ${∊}^{\left(l\right)}∼N(0,C_{∊}^{\left(l\right)})$, where $C_{∊}^{\left(l\right)}$ is the level (l) covariance matrix. The parameter vector $\theta^{\left(2\right)}$ represents the fraction of trajectory parameters carried by the covariates. A more elaborate description of this parameter vector is given in Section 7.1. In our model, we included TIV for each image as a covariate. An augmented model, $\overset{¯}{y}=\overset{¯}{X}\theta +\overset{¯}{∊}$ Eq. (11), was proposed for two levels by Friston et al. (2002) to ensure the simultaneous estimation of the hyper-parameters in a computationally efficient manner. We adopt the approach in Ziegler et al. (2015) and use Gaussian conjugate forms for the likelihood, $P(\overset{¯}{y}|\theta )$, and the prior, P(θ), providing a Gaussian posterior probability distribution Eq. (12):

$$\left\{\begin{array}{ccc} P(\theta |\overset{¯}{y}) & = & N(\theta \text{;}\eta_{\theta |y},C_{\theta |y})\\ C_{\theta |y} & = & {\left({\overset{¯}{X}}^{T}C_{∊}^{-1}\overset{¯}{X}\right)}^{-1}\\ \eta_{\theta |y} & = & C_{\theta |y}({\overset{‾}{X}}^{T}C_{∊}^{-1}\overset{¯}{y}) \end{array}\right)$$

$$\mathrm{where}\;C_{∊}=\left(\begin{array}{cc} C_{∊}^{\left(1\right)} & 0\\ 0 & C_{\theta } \end{array}\right),\;\mathrm{and}\;C_{\theta }=\left(\begin{array}{cc} C_{∊}^{\left(2\right)} & 0\\ 0 & C_{\theta }^{\left(2\right)} \end{array}\right)$$

$\eta_{\theta |y}$ and $C_{\theta |y}$ are the mean and the covariance of the Gaussian posterior distribution given the data y. The estimation is based on inferring the covariance components of a parametric empirical Bayes model at the final level through an Expectation–Maximization optimization algorithm described in Section 7.1.3.

### 2.3 Posterior Predictive Distribution

In this section, we are interested in making predictions on newly acquired values, y^, from each participant of interest at future time points. These new time points compose the subject design matrix X^. Making these predictions requires estimating the predictive probability distribution, P(y^|X^,y¯), at each voxel for the participant of interest. We propose an evaluation of P(y^|X^,y¯) given by the Eq. (1). First, the subject pooled estimate of the conditional mean, $\eta_{\theta |y}^{\left(1\right)}$, and covariance, $C_{\theta |y}^{\left(1\right)}$, are extracted from the group posterior probability using $\eta_{\theta |y}^{\left(l-1\right)}=X^{\left(l\right)}\eta_{\theta |y}^{\left(l\right)}+\eta_{∊|y}^{\left(l\right)}$ and $C_{\theta |y}^{\left(l-1\right)}=C_{∊|y}^{\left(l\right)}$ formulas at level (l) from Friston et al. (2002). Under the assumption that the likelihood and the prior are Gaussian probability distributions, the two moments previously derived are enough to build the prediction distribution Bishop (2006).

(1) $$\begin{array}{lll} P\left(\hat{y}|\hat{X},\bar{y}\right) & =\int \;d\theta \;P\left(\hat{y}|\theta ,\hat{X}\right)P\left(\theta |\hat{X},\bar{y}\right)\\ & =N\left(\hat{y}\text{;}\hat{X}\eta_{\theta |y},\sigma_{\theta |y}^{2}\right)\\ & \; & \; \end{array}$$

where σθ|y2=C∊(1)+X^Cθ|yX^T.

In order to estimate how much a new measure y^ deviates from its expected value X^ηθ|y, we integrate the area of the predictive density up to the probability score associated with the observed measure using Eq. (2). In a Gaussian representation, this integral is the error function *erf*. The *erf* is bounded in the interval [-1,1]. If the new measurement is close to the value that was associated with the highest probability, the error function will produce values around 0. On the other hand, if new measurements are far over or under the most likely value, the error function will produce, respectively, positive or negative values close to the bounding values.

(2) erfz2=y^-X^ηθ|yσθ|y2=2σθ|y2π∫0zexp-(u-X^ηθ|y)22σθ|y2du.

Given that neurodegeneration is associated with reduction in brain volume, we are interested in voxels with negative *erf* values, and those voxels where the observed value is far below the expected value are interpreted as undergoing neurodegeneration. Maps of these regions for each individual and time point are created by thresholding the *erf* maps to include voxels whose volume estimates are far from the expected value. Given that the choice of threshold may influence the sensitivity of the maps for predicting onset of symptoms, in the implementation described below that uses ADNI participants, we examined the utility of several different thresholds on prediction of dementia, ranging from *erf* values of −0.7 to −0.99.

### 2.4 Application of the Model to Empirical Data

#### 2.4.1 ADNI Cohort

In order to examine the utility of this method in a relevant context, we applied it to data from the Alzheimer’s Disease Neuroimaging Initiative (ADNI; http://adni.loni.usc.edu), which was first launched in 2003. The primary goal of ADNI has been to test whether serial magnetic resonance imaging (MRI), positron emission tomography (PET), other biological markers, and clinical and neuropsychological assessment can be combined to measure progression in elders at risk for, or suffering from early AD. ADNI participants must be at least 55 years of age and enrollees include cognitively normal individuals, people with a diagnosis of mild cognitive impairment (MCI Petersen (2016)), defined as progressive memory or other cognitive changes without significant functional impact, and patients with Alzheimer’s type dementia. Because of the long duration of ADNI, the study database contains participants who have been followed for many years. Imaging methods for ADNI have evolved over time. For this analysis, we included only those who were scanned at 1.5 Tesla on MRI scanners from one of three vendors: Philips Medical Systems, Siemens, or General Electric Medical Systems. The current analysis used the T1-weighted images, which were acquired as Magnetization Prepared Rapid Gradient Echo (MP-RAGE) images using the following parameters: 240×256×256 matrix; about 170 slices; voxel size =1.05×1.05×1.2 mm^3^; flip angle, TE and TR varied by vendor.

Because one of the proposed uses of our approach is to track the emergence and evolution of neurodegenerative changes from the earliest point possible, we elected to study people who were first assessed during a phase of normal cognition and then followed until they either eventually progressed to the point of dementia or continued to have normal cognition. We hypothesized that progression to dementia would be preceded over time by the appearance of brain regions that show unexpectedly small volume estimates based on their initial rate of change, indicating accelerated atrophy, and that the faster such regions accumulate across the brain the sooner the participant would develop dementia. Conversely, we hypothesized that individuals at high risk for dementia that did not develop dementia during the period of observation would have slower accumulation of voxels with unexpected degrees of atrophy. In ADNI, individuals at high risk can be identified based on significant accumulation of brain amyloid on PET scanning. In order to categorize patients as having or not having significant accumulation of amyloid, we used the standard uptake value ratios (SUVRs) provided by ADNI Jagust et al., 2010, Jagust et al., 2015 and applied published cutoffs appropriate for the type of scan that was acquired: 1.5 for Pittsburgh Compound B (cerebellar grey matter normalization) Jagust et al. (2010), 1.11 for AV-45 (whole cerebellum normalization) Landau et al. (2014), and 1.08 for Florbetaben (whole cerebellum normalization; https://ida.loni.usc.edu). We chose dementia as the outcome for prediction because many prior studies have shown that structural imaging can predict the onset of dementia in patients at risk Toledo et al. (2014), and dementia represents a more reliable outcome compared with MCI, because a portion of MCI patients revert to normal, indicating that their symptoms at that time might not be due to neurodegeneration Bondi et al. (2014). Symptom severity for each ADNI participant at each time point was quantified using the Clinical Dementia Rating Scale (CDR®) variable provided by ADNI Morris et al. (1997), with CDR® = 0 being cognitively normal, CDR® = 0.5 being defined as MCI, and CDR® ⩾1 being defined as dementia.

In order to model time-dependent changes in brain volume in participants of interest, we assumed that a well selected group of healthy controls have a stable rate of brain atrophy as they age, without acceleration. Under this assumption, it is a reasonable approximation to fit brain atrophy rates in a group of healthy controls using a linear model Ziegler et al. (2015), and to use this group of controls to model brain volumes in our participants of interest who were not demented for at least the first two visits after enrollment. Longitudinally studied amyloid negative individuals from ADNI who remained cognitively normal during the period of study were used as the healthy controls to model normal rates of change.

### 2.5 Illustrative Cases of Patients with FTLD

In order to illustrate the performance of the model in patients with non-AD pathology, we analyzed imaging data from two patients who were followed for several years at UCSF beginning in a phase of normal cognition and who progressed to dementia and death, and whose autopsies demonstrated FTLD pathology. These patients were studied through various research projects on FTLD and normal aging (AG019724, AG032306, AG045390, NS092089, Hillblom Network) and had MRI acquired on a Siemens Tim Trio 3 Tesla scanner using the local protocol, which acquired T1-weighted imaging using an MP-RAGE sequence using the following parameters: 160×240×256 matrix; voxel size = 1×1×1 mm^3^; flip angle 9°, TE 2.98 ms, TR 2300 ms. Clinical assessments for these studies have been described in other publications Kramer et al. (2003). The normal control dataset used for the BLME model for these cases consisted of 139 control subjects whose imaging parameters matched those of these two participants. Twenty-nine were studied at University of California San Francisco, through the projects referenced above, and an additional 110 were drawn from cognitively normal control participants in two other longitudinal studies of neurodegeneration: 34 were drawn from the Parkinson’s Progressive Markers Initiative Marek et al. (2018); 76 were cognitively normal individuals from Dominantly Inherited Alzheimer Network (DIAN McDade et al. (2018)) who did not carry dementia-causing mutations. These additional participants were included in order to have the control group span a larger age range that included potential ages of patients with FTLD, who often present at a younger age than patients with sporadic AD Olney et al. (2017). The mean age for the control group was 49.76(14.20) years old (the standard deviation is expressed between parentheses). Functional status was quantified using the CDR® for one case, but for the other we had additional data sufficient to complete the CDR® plus NACC FTLD module (CDRnFTLD) rating scale, which augments the traditional CDR® with ratings for language and behavior. Total score for the CDRnFTLD was created based on a recently published algorithm Miyagawa et al. (2020). Neuropsychological testing procedures are described in the Supplementary Materials Section 7.3, Baum et al., 1970, DELIS, 2000, Delis et al., 2001, Dempster et al., 1977, Folstein et al., 1975, Jensen, 1906, Kaplan et al., 1983, Kramer et al., 2003, Wechsler, 1997.

### 2.6 Image Processing and Quantification of Neurodegeneration

Before preprocessing of the images, all T1-weighted images were visually inspected for quality. Images with excessive motion or other artifacts were excluded. T1-weighted images underwent bias field correction using the N3 algorithm, and segmentation was performed using the SPM12 (Wellcome Trust Center for Neuroimaging, London, UK) unified segmentation Ashburner and Friston (2005). An intra-subject template was created by non-linear diffeomorphic and rigid-body registration using the symmetric diffeomorphic registration for longitudinal MRI framework Ashburner and Ridgway (2012). The intra-subject template was also segmented using SPM12’s unified segmentation. A within-subject modulation was applied by multiplying each time point’s Jacobian with the intra-subject averaged tissues Ziegler et al. (2015). A group template was generated from the within-subject average gray and white matter tissues and cerebrospinal fluid using the *Large Deformation Diffeomorphic Metric Mapping framework* Ashburner and Friston (2011). Modulated intra-subject gray and white matter were geometrically normalized and smoothed (10 mm full width half maximum Gaussian kernel) in the group template. Every step of the transformation was carefully inspected from the native space to the group template.

The modulated intra-subject gray matter density maps, normalized to the group template, for each participant were used for the BLME models and for comparison of observed with predicted values. This allowed estimation of trajectories at the voxel level, and identification of deviations from expected trajectories at time points beyond the set of initial time points used to calculate trajectory (*c.f.* methods above). For the healthy control groups used to establish the estimates of change at each voxel in the BLME model, we used all available images acquired over time in order to generate the best possible estimates. For participants of interest in whom the goal was to identify the signs of neurodegeneration as early as possible, we entered the first two images acquired at a time when they were cognitively normal into the BLME model to establish their rates of change, leaving the rest of their images for potential detection of voxels that deviate from expected volumes. This approach emulates what might be used in practice to minimize expense and time.

**Fig. 3:**
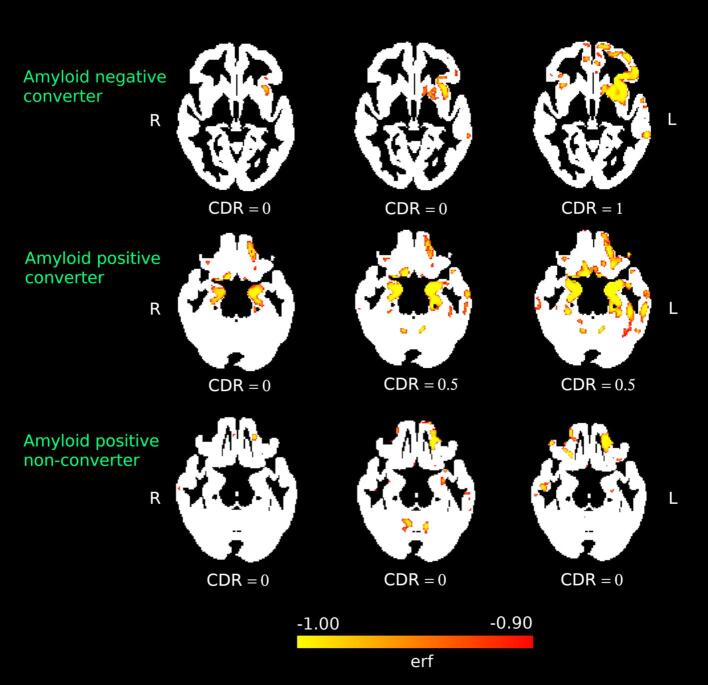
Maps of accelerated atrophy, represented as estimated *erf* value for each voxel, for three visits (columns) that occurred after the first two that were included in the BLME model, in three subjects (rows): an amyloid negative Converter (top row); an amyloid positive Converter (middle row); an amyloid positive non-converter (+ACN).

For each image that was used to compare regional gray matter density against expected values, the number of voxels showing unexpectedly low gray matter densities (identified by the *erf*, *c.f.* Fig. 3) was multiplied by the voxel size to create an atrophy cluster, quantified in cubic centimeters (*cc*). All volumetric calculations were carried out using statistical image analysis tools from the FSL Smith et al. (2004) and ANTs Avants et al. (2014) packages. To quantify the rate of spread of atrophy across the brain, we calculated the difference in size between each atrophy cluster and the atrophy cluster from the prior image and divided by the elapsed time to create a growth rate (in *cc/year*) for the period between the two scans.

Given that the goal of our analysis was to use individualized measurement of gray matter atrophy to predict conversion to dementia, we also compared the results of our method for prediction with the results of using an established individualized measure of brain atrophy that has been previously been shown to be useful for predicting onset of dementia in AD. To accomplish this, we derived estimates of bilateral hippocampal volumes (HV) using the segmentation method described above normalized in the group template and modulated to recover the participant’s hippocampal volume in native space. Regions of interest (ROI) were defined by the Desikan atlas Desikan et al. (2006), which was warped to group template space. We also calculated a rate of change for hippocampal volume (HR) by calculating the difference between each HV and the HV from the prior image. To allow comparison of effect sizes between HV, HR, cluster volume and cluster growth, the variables were converted to z-scores based on the means and standard deviations for each of these variables for all the participants of interest.

### 2.7 Statistical Analysis

Once the map of voxels affected by neurodegeneration was established for each participant, these maps were used in subsequent analysis to examine how they behaved in those who eventually converted to dementia *vs.* those who did not. To assess whether cluster growth rates were faster in patients who convert to dementia compared with those who did not convert during the period of observation, cluster volumes at different thresholds in the negative spectrum of the *erf* maps were compared between subjects that converted from cognitively normal to dementia (Converters) and amyloid positive individuals who remained cognitively normal throughout the course of study (+amyloid cognitively normal, or  + ACN). We defined the volume as the binarized map of the clusters in *cc*. Cluster volume z-scores for each participant at each acquisition time were entered into a LME analysis along with time and group *G* (Conveters vs. +ACN), and the group-by-time interaction, as predictors. Although ADNI was designed to use conversion to dementia as an outcome rather than predictor, this approach permits comparison of differences in rates of volume loss over time in those who are near conversion versus those who are not. The model is described in following equation:

(3) $$V_{\mathrm{ij}}=\alpha_{0i}+\alpha_{1i}\Delta_{j}+\varepsilon_{\mathrm{ij}}$$

where $V_{\mathrm{ij}}$ is the cluster volume for the individual i,j at the acquisition time, and $\Delta_{j}$ is the time lag between the measured cluster volume at the time *j* until the last event for that participant. For Converters, the last event was the time of conversion to dementia. For  + ACN participants, the last event was the last available observation for that individual. The vector α is the set of parameters in the model. To assess the parameters of the group-by-time interaction, we decompose the coefficient $\alpha_{1i}=\beta_{10}+\beta_{11}\times G+u_{1i}$, where $\beta_{1}$ is the parameter set and the coefficient *u* is the random effect on the parameter $\beta_{11}$ given the subject. The models were fitted using the robustlmm R package.

In order to assess the ability of spreading neurodegeneration to predict onset of dementia, the cluster volumes at different *erf* thresholds at each acquisition and the rate of cluster growth between each set of scans were used as a predictors in separate time varying Cox proportional hazard models that used time to dementia diagnosis as the survival outcome. All participants were cognitively normal at the initial time points entered into the analysis. For Converters the conversion to dementia was entered as the event at the visit where this occurred. +ACN participants were censored at their last observation. Cox regression was carried out using the lifelines package in Python (https://zenodo.org/record/3969500). Cox regressions were also run using bilateral HV and HR divided by the subject’s TIV (HVT & HRT) as predictors of dementia conversion. Because of the z-score standardization, hazard ratios (HR) estimated by the time varying Cox model can be interpreted as increasing, or reducing, the risk of dementia for each standard deviation increase in the predictor. Age and gender were used as covariates for all the Cox regressions.

## 3 Results

### 3.1 ADNI Cohort:

We were able to identify a total of 71 longitudinally studied individuals from ADNI with suitable T1-weighted images (Table 1). These included 16 Converters, 13 of whom were amyloid positive, 22  + ACN participants, and 33 amyloid negative individuals who remained cognitively normal over the period of study (- amyloid cognitively normal, or -ACN) and who served as controls for the BLME model. The -ACN participants had a mean of 5.48 images available to be entered into the BLME model.

**Table 1:** ADNI Participant Demographics.

	**Converters**	**-ACN**(†)	**+** **ACN**(††)	**All**
**N**	**16**	**33**	**22**	**71**
Age range at Baseline (min–max)	71–82	66–90	71–86	66–90
Average Baseline Age (SD) (y)	76.44 ± 3.46	72.21 ± 5.80	75.45 ± 4.24	76.96 ± 4.99
Sex (M/F)	6/10	20/13	13/9	39/32
Avg. number of scans*	4.19	3.76	5.48	3.89
Avg. time between scans (d)	357 ± 302	346 ± 286	479 ± 234	357 ± 298
Amyloid +/-	13/3	0/33	22/0	35/36

* number of scans beyond the first two entered in the BLME model, except in the case of -ACN,

for whom all images were used in the BLME model.

(†) amyloid negative individuals who remained cognitively normal throughout the period of observation.

(††) amyloid positive individuals who remained cognitively normal throughout the period of observation.

**Fig. 2:**
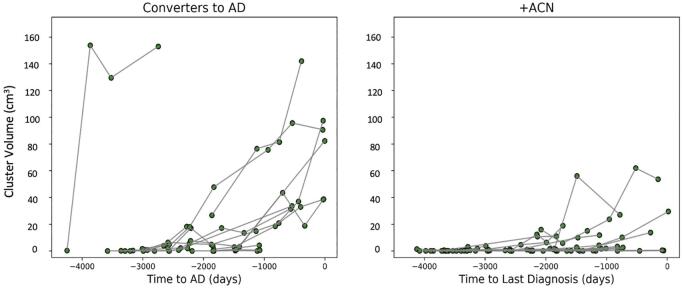
Sizes of clusters of accelerated atrophy over time in ADNI participants. The y-axis represents the cluster size (cm^3^) from the prediction model for each patient in images beyond the two time points used in the BLME model, and the x-axis represents the number of days relative to time of dementia diagnosis (for Converters), or time to last clinical diagnosis (for  + ACN), in both cases denoted as 0.

Cases in both the Converter and  + ACN groups showed the emergence of clusters of accelerated brain volume loss, with increasing cluster volume at later periods of observation (Fig. 2). We noted one individual that had a dramatic increase in cluster volume between their first and second images outside of the BLME (left panel Fig. 2). Inspection of all raw and processed images and available clinical data for this participant did not identify any meaningful differences between this individual’s data and the data from the rest of the group, so they were included in the primary analysis along with the other Converters, but we also ran the analyses excluding this participant.

The Converter group achieved higher rates of cluster volume growth, compared with the  + ACN group. The group-by-time interaction coefficient increase volume rate between Converters and +ACN was statistically significant (p<0.05), for all of the thresholds applied on the *erf* maps. A summary of the results is given in the Table 2.

**Table 2:** Cluster growth rates, in cc/year, for different thresholds applied on the *erf* maps for the Converter and the  + ACN groups cluster calculation. The values between brackets represents the confidence interval at 95%. The rate $\beta_{G*\;\Delta }$ represents the interaction rate between the group *G* and the duration Δ.

erf	−0.7	−0.8	−0.9	−0.95	−0.99	−0.999
$\beta_{G*\;\Delta }$	-1.54[-3.33,0.26]	-1.79[-3.59,0.13]	-2.05[-3.97,-0.03]	-2.18[-4.23,-0.13]	-2.43[-4.74,0.00]	-2.31[-5.00,0.26]
$\beta_{\mathrm{Converters}}$	7.04[3.46,10.38]	6.92[3.33,10.50]	6.79[2.69,10.76]	6.92[2.69,11.14]	7.43[2.56,12.30]	7.56[2.18,12.81]
$\beta_{+\mathrm{ACN}}$	4.48[2.69,6.40]	3.71[2.05,5.51]	3.07[1.28,4.74]	2.81[1.02,4.61]	2.56[0.51,4.61]	2.05[0.13,4.36]

In order to examine how this method performs in individuals with low risk for neurodegeneration, we ran a supplementary analysis where each of the -ACN controls was treated as a person of interest, using their first two images for the BLME model and looking for accelerated volume loss in their subsequent images, and this analysis produced relatively small clusters with low rates of growth over time (see supplementary materials 7.2).

In many cases, the regions where the earliest clusters were identified included the medial temporal regions, and subsequent images demonstrated spread to adjacent brain areas (Supplementary Fig. 7). However, in one case, the first cluster appeared in the left insula, and changes spread to the adjacent left frontal lobe and striatum. Although the clinical diagnosis for this case was AD and limited clinical information was available, this individual’s amyloid scan was negative. Fig. 3 shows the cluster maps in representative cases, including an amyloid positive Converter, the amyloid negative Converter discussed above, and a  + ACN case.

The cluster growth rates, but not cluster volumes, were statistically significant predictors of conversion to dementia onset in the Cox regressions for *erf* values less than −0.90, Table 3. HVT and HRT were not statistically significant predictors in the model that included Converters and  + ACN participants, although the effects were in the expected direction, with larger HVT being associated with lower likelihood of conversion at the next observation (HR  = 0.86, p  = 0.53 and CI = [0.54,1.38]) and faster HRT being associated with higher likelihood of conversion (HR  = 1.20, p  = 0.55 and CI = [0.65,2.23]). To examine whether HVT predicted dementia in a larger sample with more variability, we also ran a Cox regression that included the -ACN group. In this model, HVT became a stronger predictor of conversion, as would be expected, and the effect was statistically significant (HR  = 0.67, p  = 0.05 and CI = [0.44,1.01]). HRT was not a statistically significant predictor in this model (HR  = 0.93, p  = 0.82 and CI = [0.49,1.75]).

**Table 3:** Hazard ratio at different threshold of the *erf* map in the negative spectrum. The hazard ratio (HR) is expressed with the confidence interval at 95% between brackets. Size indicates the volume values; Rate indicates the temporal variation of the two metric volume. Bold values are statistically significant at p<0.05. The lines noted with O present the statistics including the outlier; lines with N present the statistics without the outlier.

*erf* thr.		−0.7	−0.8	−0.9	−0.95	−0.99	−0.999
HR Size	O	1.26[0.87–1.82]	1.24[0.88–1.76]	1.23[0.88–1.70]	1.22[0.89–1.68]	1.23[0.91–1.67]	1.25[0.92–1.67]
	N	**2.02[1.25–3.27]**	**1.95[1.26–3.01]**	**1.82[1.25–2.66]**	**1.72[1.22–2.43]**	**1.55[1.13–2.11]**	**1.42[1.05–1.92]**
HR Rate	O	1.93[0.79–4.75]	2.13[0.89–5.09]	**2.34[1.05–5.21]**	**2.43[1.17–5.07]**	**2.15[1.19–3.87]**	**1.81[1.06–3.11]**
	N	1.57[1.09–3.47]	1.68[0.92–3.07]	**1.86[1.06–3.26]**	**2.04[1.183.52]**	**2.09[1.093.65]**	**1.95[1.16–3.30**

We also re-ran these Cox regressions after excluding the participant with the unusual pattern of change in left panel of Fig. 2. In these analyses both cluster volume and cluster growth rate were significant predictors of conversion, Table 3. HVT and HRT were not statistically significant predictors in these models, but all estimated effects were similar in magnitude to the estimates in the main Cox regression and in the correct directions (HR  = 0.84, p  = 0.46 and CI = [0.52,1.34] for HVT, and HR  = 1.16, p  = 0.62 and CI = [0.63,2.14] for HRT).

We were also interested in whether the clusters detected with our approach could have predictive value early in the evolution of disease, even before mild symptoms developed. We therefore ran a survival analysis where any observations collected at a time when a participant was diagnosed with MCI were excluded. We had to remove two Converters from the model looking at cluster growth rate because they had only one observation as a normal participant outside of the two observations used for the BLME. Cluster volume was a statistically significant predictor of eventual dementia onset in this model, and the hazard ratio for cluster growth was close to being statistically significant (volume: HR  = 1.52, p  = 0.05 and CI = [1.00,2.33]; growth: HR  = 1.72, p  = 0.06 and CI = [0.98, 3.00]). HVT and HRT were not statistically significant predictors (HR  = 1.47, p  = 0.32 and CI = [0.69,3.15] for the HVT; HR  = 1.83, p  = 0.16 and CI = [0.79,4.24] for HRT), even when -ACN participants were included (HR  = 1.26, p  = 0.59 and CI = [0.55,2.84] for HVT; HR  = 1.76, p  = 0.22 and CI = [0.71,4.37] for HRT).

### 3.2 FTLD Cases:

**Fig. 4:**
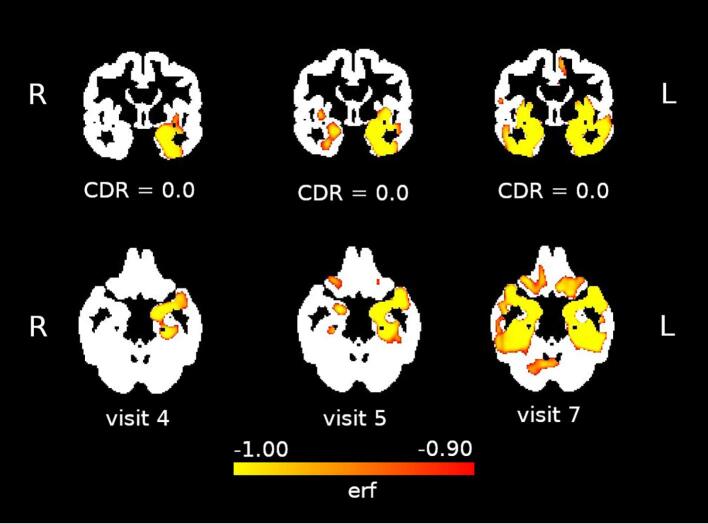
Case 1 – Regions of accelerated atrophy over three visits (see Table 4).

*Case* *1:*This participant was initially enrolled at the age of 85 in a study of normal aging at our center, and clinical history, exam and cognitive testing confirmed that they were cognitively normal for their age (see Table 4 for selected scores). At the seventh visit, they complained of mild word finding difficulties, such as names of famous people, and also trouble with details of events from a movie they had seen a few days earlier, which they said had been worsening over the prior year-and-a-half. At their 8th visit, the participant complained that their memory had “plummeted downhill” during the prior year. Over the next few months, the participant’s memory continued to decline and they progressively stopped eating, and they died about eight months after the last assessment. Amyloid scans in their first and seventh visits were both negative. Autopsy revealed a gross pattern of left greater than right temporal lobe atrophy. Microscopic examination revealed gliosis that was most prominent in the left entorhinal cortex, amygdala and inferior temporal gyrus, and there were numerous cortical neuritic inclusions, and long TDP-43 positive dystrophic neurites, consistent with a primary pathological diagnosis of TDP-43 Type C pathology Mackenzie et al. (2009). Incidental AD type pathology, with moderate amyloid plaque burden and neurofibrillary tangles limited to the entorhinal cortex (Braak Stage 1), was also identified. Case 1 had five MRIs available for analysis. The first was associated with visit 1, the second was obtained at a visit for an experimental imaging protocol without clinical data, and the rest were obtained at visits 4, 5 and 7 (Table 4). The first two images were used to model volume changes over time using the BLME framework. The subsequent images, thresholded at an *erf* of −0.9, demonstrate expanding regions of accelerated atrophy, beginning in the left anterior medial temporal and inferior temporal regions in the earliest post-model images, and expanding to adjacent temporal regions and to contralateral temporal regions over time (Fig. 4).

**Table 4:** Case 1 – medical assessment per visit.

		**Visit 1**	**Visit 2**	**Visit 3**	**Visit 4**	**Visit 5**	**Visit 6**	**Visit 7**	**Visit 8**
**Years from Initial Visit**		0.00	0.75	1.42	2.59	3.17	4.08	5.42	6.42
**Domain**	**Measure**								
General Cognition	MMSE	29	-	29	28	28	27	27	24
Memory	CVLT-Long Delay (16 word list)	12	-	10	12	10	5	-	-
	Benson Recall (max 17)	13	-	-	13	15	12	16	8
Frontal/Executive	Digits BW	6	-	-	6	-	5	-	5
	Phonemic Fluency	12	-	-	17	15	8	-	15
	Category Fluency	24	-	-	25	19	18	13	10
Visuospatial	Benson Copy (max 17)	16	-	-	17	15	16	16	13
Language	Boston Naming Test	15	-	15	15	14	12	12	7
Function (CDR)*	Memory	0	-	0	0	-	0	0.5	0.5
	Orientation	0	-	0	0	-	0	0	0
	Judgment and Problem Solving	0	-	0	0	-	0	0	0
	Community Affairs	0	-	0	0	-	0	0	0.5
	Home and Hobbies	0	-	0	0	-	0	0	0
	Personal Care	0	-	0	0	-	0	0	0
	CDR Total Rating	0	-	0	0	-	0	0.5	0.5
Imaging	MRI	X**	X**		X	X		X	
	Amyloid PET	X						X	

* Details in Supplementary Materials Section 7.3.

** Image used in BLME Model.

**Fig. 5:**
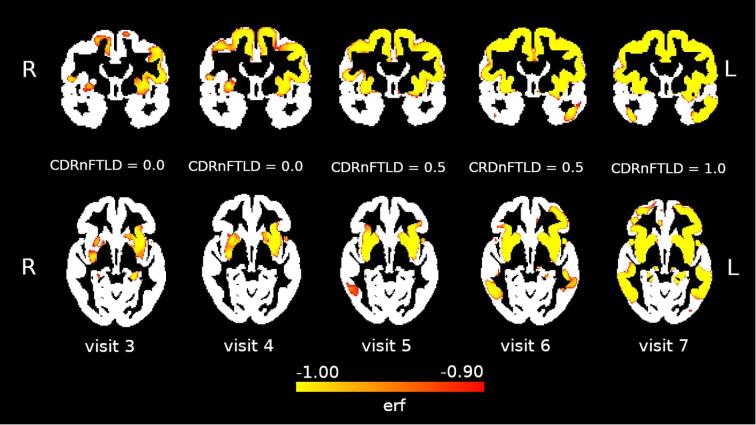
Case 2 – Regions of accelerated atrophy over four visits (see Table 5).

*Case* *2:*This participant was initially enrolled at age 65 in a study of FTLD at our center because of a family history of FTLD in siblings and a parent. Genetic testing revealed a pathogenic *GRN* variant Ramos et al. (2020). At the initial assessment, the participant complained of mild, occasional word finding trouble that was not having any impact on function, and the remainder of the history, exam, and cognitive testing confirmed normal cognitive and behavioral function (Table 5). Over the next three visits, they complained of stable, mild word finding trouble, not noted by others in their family, and their cognitive performance remained stable, except for some trouble enunciating words during cognitive testing at the fourth visit (suggesting possible apraxia of speech). At the fifth visit, the participant endorsed significant changes in language over the prior year, including “stumbling” over words, particularly those that are difficult to pronounce. The examination at that visit showed fragmented, effortful speech with pronunciation errors and good comprehension, consistent with apraxia of speech and non-fluent progressive aphasia. At subsequent visits, the speech output difficulties had worsened and the participant developed parkinsonian features, particularly on the left side, and the speech difficulties forced them to stop work by the seventh visit, at which point they were nearly mute. At the last visit, cognitive changes had extended to executive function by history, and physical function had declined significantly. Formal cognitive testing could not be completed. The participant continued to decline at home and died two years after the last research visit. Autopsy identified frontal, anterior temporal, anterior parietal, striatal and thalamic atrophy. Microscopic examination revealed gliosis that was most prominent in the frontal and parietal cortex, striatum and thalamus, and large numbers of TDP-43 neuronal cytoplasmic inclusions in cortical and subcortical regions, more abundant in the upper cortical layers, which is consistent with FTLD TDP-43 type A pathology Mackenzie et al. (2009). Incidental AD pathology in the form of moderate neuritic plaque burden and mild neurofibrillary tangle pathology (Braak Stage 2) was also identified. Case 2 had seven MRIs available for analysis. The first two were associated with visits 1 and 2 in Table 5, and were used to model volume changes over time using the BLME framework. The subsequent images correspond to visits 3 through 7. These images, thresholded at an *erf* of −0.9, demonstrate expanding regions of accelerated atrophy, beginning in the left greater than right insula and operculum, along with involvement of the dorsal medial frontal region, and expanding to include bilateral insula, operculum, striatum, dorsal and medial frontal cortex and temporal lobes by visit 7 (Fig. 5).

**Table 5:** Case 2 – medical assessment per visit.

		**Visit 1**	**Visit 2**	**Visit 3**	**Visit 4**	**Visit 5**	**Visit 6**	**Visit 7**	**Visit 8**
**Years Since Initial Visit**		0.00	0.92	1.92	2.92	3.92	4.84	5.84	6.92
**Domain**	Measure*								
General Cognition	MMSE	28	30	30	29	30	29	23	-
Memory	CVLT-SF-Long Delay (9 word list)	9	9	9	9	9	9	9	-
	Benson Recall (max 17)	11	12	10	11	12	9	9	-
Frontal/Executive	Digits BW	4	4	3	5	5	3	4	-
	Phonemic Fluency	31	23	27	33	23	23	16	-
	Category Fluency	40	37	37	42	33	27	12	-
Visuospatial	Benson Copy (max 17)	16	16	16	16	16	15	13	-
Language	Boston Naming Test	15	15	15	15	15	15	15	-
Function (CDRnFTLD)	Memory	0	0	0	0	0	0	0	0
	Orientation	0	0	0	0	0	0	0	0.5
	Judgment and Problem Solving	0	0	0	0	0	0.5	0.5	1
	Community Affairs	0	0	0	0	0	0	0	2
	Home and Hobbies	0	0	0	0	0	0	0	2
	Personal Care	0	0	0	0	0	0	0	2
	Language	0	0	0	0	1	1	2	2
	Behavior	0	0	0	0	0	0.5	0.5	0.5
	CDR Total Rating	0	0	0	0	0	0	0	0.5
	CDRnFTLD Total Rating	0	0	0	0	0.5	0.5	1	2
Imaging	MRI	X**	X**	X	X	X	X	X	

* Details in Supplementary Materials Section 7.3.

** Image used in BLME model.

## 4 Discussion

In the present study, we have described the use of a Bayesian longitudinal modeling framework to identify brain regions that are undergoing accelerated loss of brain volume, indicating neurodegeneration, in individual cases, using only two initial images to model baseline rates of change for each participant of interest. Application of this model to cognitively normal individuals, some of whom eventually developed dementia, identified focal regions of neurodegeneration either before or accompanying the earliest symptoms. Continued longitudinal observation revealed expansion of neurodegeneration to contiguous brain regions as well as distant regions, including homologous locations in the opposite hemisphere. Spread of these regions across the brain was more rapid in patients that ultimately converted to dementia compared with those who remained cognitively normal during extended observation, and the rate of this spread was a statistically significant predictor of the likelihood of developing dementia. Lastly, the brain regions where these abnormalities appeared varied across people, including early involvement of insular, frontal, and striatal regions in patients who had proven, or likely non-AD pathologies.

This approach has many potential uses for research and care of patients with neurodegenerative disease. The application we examined, prediction of dementia onset, can be used in intervention trials designed to prevent onset of symptoms by helping with selection of patients more likely to develop symptoms within the proposed duration of the study, improving efficiency Boxer et al., 2020, Rosen et al., 2020b. Once effective preventative treatments have been developed, patients at elevated risk of neurodegenerative disease, based on monogenic, polygenic, or other biomarker profiles, can be tracked using this technique to identify when the risk is beginning to manifest in early signs of neurodegeneration, and treatment can be initiated. Beyond the application to prediction of dementia onset, the same method of identifying voxels that are likely to be undergoing neurodegeneration can be used in other types of analyses. For instance, the changes detected using this approach can also be used to track the effects of treatment in multiple ways. One approach would be to compare the rate of spread in placebo versus treatment groups, which would be similar to our analysis comparing converters with non-converters, only with à priori grouping based on treatment assignment. The method could also be used to track individualized responses to treatment. Once the rates of change in each region and spread across the brain have been quantified in an individual, the predictions from this algorithm could be used to identify regions that have better than expected values (*e.g.* higher gray matter content than expected), which may be used to support a response to treatment Frost et al. (2008). Both of these approaches can be used in trials with symptomatic patients, but may be particularly important in prevention trials, when symptoms cannot be used to detect the effect of an intervention, increasing the importance of biomarker outcomes Rosen et al. (2020b). The estimates generated by this method can also be used for other types of predictions besides time to dementia. For instance, the maps of neurodegeneration can be compared with known patterns of atrophy associated with different dementia syndromes in order to predict what symptoms a person might develop, or to interpret the meaning of mild changes in cognitive performance in a person that has thus far been asymptomatic. Each of these applications could use the same approach for estimation of voxel health, but would use a different approach in place of our Cox regression in order to address the question of interest. Furthermore, the utility of this technique extends well beyond measures of brain volume. Current models of neurodegenerative disease stipulate that many biomarkers and clinical effects of these disorders enter a phase of accelerated change Jack et al. (2013). For many of these measurements, the power of this technique to identify a subset of observations (*e.g.* from among many voxels, body fluid measurements, or cognitive tests) using a reproducible threshold without à priori assumptions could provide increased sensitivity to early neurodegenerative change in diverse groups of patients.

The majority of participants included in this analysis were from ADNI, and therefore likely to have AD pathology and neurodegeneration beginning in the medial temporal region, which was true for most of the cases in this study. One case from the ADNI cohort, however, along with two cases from the UCSF cohort, reinforce the sensitivity of this technique to the effects of pathologies and clinical presentations beyond typical AD. An ADNI case showed the emergence of changes in the left insular region that progressed to involve frontal cortex and striatum. The pattern of brain atrophy from this case is typical for the non-fluent variant of primary progressive aphasia (nfvPPA) Gorno-Tempini et al. (2004). The existence of this case is consistent with prior work that highlighted the presence of participants with suspected non-AD pathology in ADNI Caroli et al. (2015). Although the reported clinical diagnosis in this case was AD, this participant’s amyloid scan was negative, and there is a reasonably high likelihood that this case had FTLD pathology. Case 1 from UCSF showed the emergence of left anterior and inferior temporal changes, which was consistent with the participant’s early word finding and episodic memory complaints. The final pathological diagnosis was TDP-43 Type C, which is typically associated with the semantic variant of PPA and manifests with early atrophy in the left anterior temporal and inferior temporal region Seeley et al. (2005), consistent with results from the BLME model. This patient may have been just entering a phase of significant semantic loss before they died, as indicated by significant drops in picture naming and category fluency scores at their last visit (more targeted assessments of semantic processing were not available). Case 2 showed early left greater than right insular and frontal opercular changes, which is consistent with the signs of speech apraxia that were noted at the next visit, and prior studies localizing speech apraxia to the left insular region Dronkers (1996). Case 2 subsequently developed a full nfvPPA syndrome, which has been localized to left frontal opercular region in prior studies Gorno-Tempini et al. (2004). Furthermore, the identification of dorsomedial frontal changes in Case 2 is consistent with prior observations indicating that spread to this region occurs nfvPPA Mandelli et al. (2016). These observations support the idea that this technique is sensitive to the emergence of neurodegeneration due to a variety pathologies and affecting diverse neural systems. In patients at risk for AD, the technique may allow the detection of degeneration even in patients with young onset and atypical syndromes, which are often associated with prominent involvement of frontal and parietal, rather than medial temporal regions Ossenkoppele et al. (2015). In FTLD, the same pathology can present with several clinical syndromes, and no current methods allow prediction of which one will occur in a given person Olney et al. (2017). Ongoing studies of FTLD due to autosomal dominant mutations Rohrer et al., 2015, Rosen et al., 2020a will provide the opportunity to more formally assess the sensitivity and predictive value of this technique for specific syndromes as more patients within these cohorts develop symptoms Chen et al., 2019, Chen et al., 2020. Similar questions also arise in patients at risk for synucleinopathies, where the emergence of Parkinson’s disease and diffuse Lewy body syndromes are important to detect as early as possible Marek et al. (2018). In all of the cases studied, the anatomical changes were identified at a time when there were either no symptoms or very mild symptoms of questionable significance, suggesting that this technique can have great value in helping to decide when mild symptoms are an indicator of neurodegeneration versus not.

The purpose of this initial report is primarily a description of this method and demonstration of potential utility. There are many possible modifications and expansions that should be considered in order to develop this technique for maximum value. For instance, we used only two images for the BLME to estimate the initial rates of change, but if larger datasets with more time points in a period of normal cognition become available, it may be possible to examine the value of more time points for the initial estimates. Similarly, our model assumed linear change at baseline, but studies have identified non-linear components of change even in normal aging Schuff et al. (2012). Exploration of non-linear components in our models may improve the sensitivity and specificity of detection, and use of more than two images in the BLME model would be important for this assessment. Our analysis also indicates that, although this method does not appear to identify many voxels in people who are unlikely to be experiencing neurodegeneration (see supplemental materials 7.2), the choice of threshold for defining voxels of neurodegeneration may have some impact on the utility of the technique for predicting symptom onset. Selection of a threshold could be optimized to empirically identify the thresholds that are best for the intended use of the procedure. In the example above the outcome of symptom onset time could be used, but this would best be accomplished when larger datasets that include more observed conversions become available. The resulting optimized threshold might be different than if the technique was used for other purposes such as tracking of treatment effects or prediction of types of symptoms. The choice of control group is also an important consideration. We chose the -ACN group because, for studies seeking to replicate our findings, it would be easier to assemble such a group than to identify a  + ACN group with similar numbers of imaging and clinical features. Also, our analysis tested the value of our model under the assumption that any amyloid positive individual could potentially develop dementia, which is what must be assumed when a participant is first entered into a study or a patient is evaluated in a clinic. That said, use of an appropriately representative  + ACN group that has been followed for several years without progression to dementia might ultimately improve the model’s predictive value. Expansion of this method to account for multiple modes of imaging simultaneously, as well as additional biomarkers, offers additional opportunities.

Lastly, the utility of this approach must be considered in light of existing measures with proven utility in predicting and tracking symptoms. Although in this small dataset, our method was a more useful predictor of dementia than HV, there are many studies showing that HV, volumetric measurements of other brain regions, and many other biomarkers, have value in predicting onset of symptoms, in both AD Weiner et al. (2017) and other dementias Staffaroni et al. (2020a). Prior studies have established the value of HV for predicting dementia in larger cohorts (> 100 cases) of individuals with MCI who were amyloid positive Jack et al., 2010, van et al., 2012, Ten et al., 2017, Jang et al., 2019. The effect size for the cluster growth rate in our study (100% change in HR for each standard deviation) was larger than the effect size for HV (about 15% change in HR). In addition, our study suggested that cluster growth may have predictive value in asymptomatic people. However, larger studies would need to be done to better establish whether this method for detection of neurodegeneration is more valuable for prediction of dementia in AD compared with established measures such as HV, to establish the magnitude of the improved prediction, and to make this comparison in other types of dementia where the value of à priori regions of interest are less well established. Furthermore, several studies have used deep learning and related techniques to characterize various patterns of brain abnormalities associated with established dementia syndromes Weiner et al., 2017, Rathore et al., 2017, Young et al., 2018. These types of methods can potentially be used to interpret emerging patterns detected with the technique described here in order to improve prediction of both what specific symptoms to expect and when those symptoms will develop.

## 5 Conclusion

BLME models designed to identify brain regions that depart from their expected trajectory appear to be a promising method for detecting the emergence of neurodegeneration in normal individuals at increased risk for neurodegenerative disease. The initial application of this approach suggests that it is useful for detecting changes due to a variety of pathologies in multiple neurological systems, and that the speed at which these changes spread across the brain can be used to predict onset of dementia. In addition, the location where the changes are identified might be useful for predicting which symptoms an individual will develop first, and for confirming when very mild symptoms are due to neurodegeneration. Further work to refine and expand this technique is required to define its best use for intervention trials and for management of neurodegenerative diseases.

## CRediT authorship contribution statement

**Yann Cobigo:** Writing - original draft, Formal analysis, Conceptualization, Methodology, Software, Writing - review & editing. **Matthew S. Goh:** Data curation. **Amy Wolf:** Data curation. **Adam M. Staffaroni:** Investigation. **John Kornak:** Writing - review & editing. **Bruce L. Miller:** Investigation. **Gil D. Rabinovici:** Investigation. **William W. Seeley:** Investigation. **Salvatore Spina:** Investigation. **Adam L. Boxer:** Investigation. **Bradley F. Boeve:** Investigation. **Lei Wang:** Writing - review & editing. **Ricardo Allegri:** Investigation. **Marty Farlow:** Investigation. **Hiroshi Mori:** Investigation. **Richard J. Perrin:** Investigation. **Joel Kramer:** Investigation. **Howard J. Rosen:** Writing - original draft, Methodology, Supervision, Conceptualization, Writing - review & editing.

## Declaration of Competing Interest

The authors declare that they have no known competing financial interests or personal relationships that could have appeared to influence the work reported in this paper.
