# Supplementary material for: Detection of emerging neurodegeneration using Bayesian linear mixed-effect modeling
Source: Neuroimage Clin. 2022 Aug 6;36:103144. doi: 10.1016/j.nicl.2022.103144 (PMC9428846; doi:10.1016/j.nicl.2022.103144)
Supplement: Supplementary data 1 [file mmc1.pdf]

7. Supplementary Material

7.1. Estimating anatomical trajectories with Bayesian mixed-effects modeling

This section presents a detailed description of the application of a Bayesian linear mixed-effects model with a hierarchical data structure to neuroimaging data at the voxel level. In many fields, including neuroimaging, mixed-effect models offer a flexible approach to unbalanced data, sparse matrices, and variable timing of follow-up. This section mostly summarizes the description in Ziegler et al. Ziegler et al. (2015), and is provided here to permit interpretation of our findings without necessarily consulting that paper. Some important equations from Friston et al. Friston et al. (2002) were added to help readers to follow all of the steps. We encourage the audience to review the original publications for more detailed explanations.

7.1.1. Hierarchical model

The proposed model is a generative model with a hierarchical structure. The authors used a time-dependent polynome of degree D: $y_{ij} = \sum_{d=0}^{D} \theta_{id}^{(1)} t_j^d + \epsilon_{ij}^{(1)}$ for the first level of the hierarchical structure, where y_{ij} is the response (modality outcome in a voxel) for the subject i at the acquisition j. The canonical base of the polynome is built from the polynomes $[t_j^0, t_j^1, \ldots, t_j^D]$ with t_j representing the age of the subject at the acquisition point j. The vector $\boldsymbol{\theta}_i^{(1)}$ and $\epsilon_{ij}^{(1)}$ are, respectively, the first level vector of parameters and noise. The complete model is written in the following compact way: $\mathbf{y} = \mathbf{X}^{(1)}\boldsymbol{\theta}^{(1)} + \boldsymbol{\epsilon}^{(1)}$, with $\mathbf{X}^{(1)}$ and $\boldsymbol{\epsilon}^{(1)}$, respectively, being the first level design matrix built from the subject's age t_j at the j-th time of image acquisition and a noise vector. A more explicit version of the first level linear model is given by eq. (4) for N subjects.

$$\begin{pmatrix} \mathbf{y}_1 \\ \mathbf{y}_2 \\ \vdots \\ \mathbf{y}_N \end{pmatrix} = \begin{pmatrix} \mathbf{X}_1^{(1)} & & & \\ & \mathbf{X}_2^{(1)} & & \\ & & \ddots & \\ & & & \mathbf{X}_N^{(1)} \end{pmatrix} \begin{pmatrix} \boldsymbol{\theta}_1^{(1)} \\ \boldsymbol{\theta}_2^{(1)} \\ \vdots \\ \boldsymbol{\theta}_N^{(1)} \end{pmatrix} + \boldsymbol{\epsilon}^{(1)} \tag{4}$$

The second level is modeled with $\boldsymbol{\theta}^{(1)} = \mathbf{X}^{(2)}\boldsymbol{\theta}^{(2)} + \boldsymbol{\epsilon}^{(2)}$, where $\mathbf{X}^{(2)}$, $\boldsymbol{\theta}^{(2)}$ and $\boldsymbol{\epsilon}^{(2)}$ are, respectively, the second level design matrix composed from baseline covariates (biomarkers, neuroimages global metrics, ...), parameters and a noise vector. The second level design matrix is written in a compact way, using the Kronecker product: $\mathbf{X}^{(2)} = [[J_{N,1}\ Z] \otimes I_{D+1}]$, where $J_{N,1}$ is a column of ones, I_{D+1} is the $[(D+1)\times(D+1)]$ dimension identity matrix and Z is a $[N \times R]$ dimension covariate matrix of N subjects having R covariates each. The system (5) summarizes the dependence between the levels Ziegler et al. (2015):

$$\begin{aligned} \mathbf{y} &= \mathbf{X}^{(1)}\boldsymbol{\theta}^{(1)} + \boldsymbol{\epsilon}^{(1)} \\ \boldsymbol{\theta}^{(1)} &= \mathbf{X}^{(2)}\boldsymbol{\theta}^{(2)} + \boldsymbol{\epsilon}^{(2)} \\ &\vdots \\ \boldsymbol{\theta}^{(n-1)} &= \mathbf{X}^{(n)}\boldsymbol{\theta}^{(n)} + \boldsymbol{\epsilon}^{(n)} \end{aligned} \tag{5}$$

The error distribution, for the level (i), is i.i.d Gaussian noise $\boldsymbol{\epsilon}^{(i)} \sim \mathcal{N}(\mathbf{0}, \mathbf{C}_\epsilon^{(i)})$ with zero mean and covariance $\mathbf{C}_\epsilon^{(i)}$. The level momenta can be deduced from the following structure:

$$\begin{aligned} E[\boldsymbol{\theta}^{(i)}] &= \boldsymbol{\eta}_\theta^{(i)} = \mathbf{X}^{(i+1)}\boldsymbol{\eta}_\theta^{(i+1)} \\ E[(\boldsymbol{\theta}^{(i)} - \boldsymbol{\eta}_\theta^{(i)})(\boldsymbol{\theta}^{(i)} - \boldsymbol{\eta}_\theta^{(i)})^T] &= \mathbf{C}_\theta^{(i)} = E[\boldsymbol{\epsilon}^{(i+1)}\boldsymbol{\epsilon}^{(i+1)T}] = \mathbf{C}_\epsilon^{(i+1)} \end{aligned} \tag{6}$$

In the Bayesian framework, the model (5) can be written recursively in the following manner:

$$\begin{aligned} \mathbf{y} &= \boldsymbol{\epsilon}^{(1)} + \mathbf{X}^{(1)}\boldsymbol{\epsilon}^{(2)} + \cdots + \mathbf{X}^{(1)} \cdots \mathbf{X}^{(n-1)}\boldsymbol{\epsilon}^{(n)} \\ &+ \mathbf{X}^{(1)} \cdots \mathbf{X}^{(n)}\boldsymbol{\theta}^{(n)} \\ &= \mathbf{X}\boldsymbol{\theta} + \boldsymbol{\epsilon}^{(1)} \end{aligned} \tag{7}$$

Using a compact structure for the design matrices $\mathbf{X} = [\mathbf{X}^{(1)}, \mathbf{X}^{(1)}\mathbf{X}^{(2)}, \cdots, \mathbf{X}^{(1)}\mathbf{X}^{(2)}\cdots\mathbf{X}^{(n)}]$ and $\boldsymbol{\theta} = [\boldsymbol{\epsilon}^{(2)}, \cdots, \boldsymbol{\epsilon}^{(n)}, \boldsymbol{\theta}^{(n)}]^T$. Eq. (7) is used to extract the full hierarchical model momenta:

$$
\begin{aligned}
E\{\mathbf{y}\mathbf{y}^T\} &= \underbrace{\mathbf{C}_\epsilon^{(1)}}_{error} + \underbrace{\mathbf{X}^{(1)}\mathbf{C}_\epsilon^{(2)}\mathbf{X}^{(1)T}}_{random\ effects\ level\ 2} + \cdots \\
&+ \underbrace{\mathbf{X}^{(1)}\cdots\mathbf{X}^{(i-1)}\mathbf{C}_\epsilon^{(i)}\mathbf{X}^{(i-1)T}\cdots\mathbf{X}^{(1)T}}_{random\ effects\ level\ i} \\
&+ \cdots + \underbrace{\mathbf{X}^{(1)}\cdots\mathbf{X}^{(n)}\mathbf{C}_\theta^{(n)}\mathbf{X}^{(n)T}\cdots\mathbf{X}^{(1)T}}_{fixed\ effects} \\
&= \mathbf{C}_\epsilon^{(1)} + \mathbf{X}\mathbf{C}_\theta\mathbf{X}^T
\end{aligned}
\tag{8}
$$

where

$$
Cov\{\boldsymbol{\theta}\} = \mathbf{C}_\theta = \begin{pmatrix} \mathbf{C}_\epsilon^{(2)} & \cdots & \mathbf{0} & \mathbf{0} \\ \vdots & \ddots & \vdots & \vdots \\ \mathbf{0} & \cdots & \mathbf{C}_\epsilon^{(n)} & \mathbf{0} \\ \mathbf{0} & \cdots & \mathbf{0} & \mathbf{C}_\theta^{(n)} \end{pmatrix}
\tag{9}
$$

and

$$
E\{\boldsymbol{\theta}\} = \boldsymbol{\eta}_\theta = \begin{pmatrix} 0 \\ \vdots \\ 0 \\ \boldsymbol{\eta}_\theta^{(n)} \end{pmatrix}
\tag{10}
$$

This Bayesian formulation uses implicit empirical prior covariance components. In other words, the highest level of the covariant component will be set to infinity: $\mathbf{C}_\theta^{(n)} = \infty$. The original authors used an augmented model ensuring the covariance hyper-parameters (described later in section 7.1.2) will be fitted at the same time as the model parameters.

$$
\bar{X}\boldsymbol{\theta} = \begin{pmatrix} \mathbf{X}^{(1)}, & \mathbf{X}^{(1)}\mathbf{X}^{(2)}, & \cdots, & \mathbf{X}^{(1)}\mathbf{X}^{(2)}\cdots\mathbf{X}^{(n)} \\ I & \mathbf{0} & \cdots & \mathbf{0} \\ \mathbf{0} & I & \cdots & \mathbf{0} \\ \vdots & & & \vdots \\ \mathbf{0} & \cdots & \mathbf{0} & I \end{pmatrix} \begin{pmatrix} \boldsymbol{\epsilon}^{(2)} \\ \vdots \\ \boldsymbol{\epsilon}^{(n)} \\ \boldsymbol{\theta}^{(n)} \end{pmatrix}
$$

The augmented hierarchical model equations and their associated distributions become

$$
\begin{aligned}
\bar{\mathbf{y}} &= \bar{X}\boldsymbol{\theta} + \bar{\boldsymbol{\epsilon}} \\
\begin{pmatrix} \mathbf{y} \\ \boldsymbol{\eta}_\theta \end{pmatrix} &= \bar{X}\boldsymbol{\theta} + \begin{pmatrix} \boldsymbol{\epsilon}^{(1)} \\ \boldsymbol{\eta}_\theta - \boldsymbol{\theta} \end{pmatrix}
\end{aligned}
\tag{11}
$$

$$
p(\bar{\mathbf{y}}|\boldsymbol{\theta}) = \mathcal{N}(\bar{\mathbf{y}}; \bar{X}\boldsymbol{\theta}, \mathbf{C}_\epsilon) \ \ and \ \ p(\boldsymbol{\theta}) = \mathcal{N}(\boldsymbol{\theta}; \boldsymbol{\eta}_\theta, \mathbf{C}_\theta)
$$

$$
\boldsymbol{\eta}_\theta = \begin{pmatrix} \mathbf{0} \\ \boldsymbol{\eta}_\theta^{(2)} \end{pmatrix}, \ \mathbf{C}_\epsilon = \begin{pmatrix} \mathbf{C}_\epsilon^{(1)} & \mathbf{0} \\ \mathbf{0} & \mathbf{C}_\theta \end{pmatrix}
$$

We assume our data are distributed with a Gaussian distribution and our parameters, at each level, are also à priori distributed in a Gaussian manner. Since the likelihood and the parameters à priori are Gaussian distributed, the product of two Gaussians gives a Gaussian. In other words, the posterior distribution of the parameters will also be a Gaussian distribution conjugate to the parameter prior distribution. To compute the posterior momenta, we use the Bayes rule $p(\boldsymbol{\theta}|\bar{\mathbf{y}}) = p(\bar{\mathbf{y}}|\boldsymbol{\theta})p(\boldsymbol{\theta})/p(\bar{\mathbf{y}})$. The posterior mean and covariance for the parameters are:

$$\begin{aligned} \mathbf{C}_{\theta|y}^{-1} &= \bar{\mathbf{X}}^T \mathbf{C}_\epsilon^{-1} \bar{\mathbf{X}} \\ \boldsymbol{\eta}_{\theta|y} &= \mathbf{C}_{\theta|y} \left(\bar{\mathbf{X}}^T \mathbf{C}_\epsilon^{-1} \bar{\mathbf{y}} \right) \end{aligned} \tag{12}$$

Where

$$\begin{aligned} \mathbf{C}_\epsilon &= \begin{pmatrix} \mathbf{C}_\epsilon^{(1)} & \mathbf{0} \\ \mathbf{0} & \mathbf{C}_\theta \end{pmatrix} \\ \bar{\mathbf{X}} &= \begin{pmatrix} \mathbf{X} \\ I \end{pmatrix} \\ \bar{Y} &= \begin{pmatrix} Y \\ \boldsymbol{\eta}_\theta \end{pmatrix} \end{aligned}$$

7.1.2. Covariance estimation

The parameters can be inferred by estimating the covariance components. The covariance components must be estimated at every level. We can use the iterative procedure, using the error covariances as priors $\mathbf{C}_\theta^{(i-1)} = \mathbf{C}_\epsilon^{(i)}$, eq. (6). We use $\mathbf{C}_\epsilon^{(i)} = \sum_j \lambda_j^{(i)} Q_j^{(i)}$, where $\lambda_j^{(i)}$ are the hyper-parameters and $Q_j^{(i)}$ represent some bases set for the covariance matrix. The bases can be constructed as a constraint on the prior covariance structures in the same way as the $\mathbf{X}^{(i)}$ specify constraints on the prior expectation. $Q_j^{(i)}$ embodies the form of the jth component at the ith level and models different variances for different levels and different forms of correlations within the levels. A linear decomposition of $\mathbf{C}_\epsilon^{(i)}$ is a natural parametrization because the different sources of conditionally independent variance add linearly and the constraints can be specified directly in terms of these components $\mathbf{C}_\epsilon = \mathbf{C}_{\theta,(n)} + \sum_k \lambda_k Q_k$.

$$\mathbf{C}_{\theta,(n)} = \begin{pmatrix} \mathbf{0} & \cdots & \mathbf{0} & \mathbf{0} \\ \vdots & \ddots & \vdots & \vdots \\ \mathbf{0} & \cdots & \mathbf{0} & \mathbf{0} \\ \mathbf{0} & \cdots & \mathbf{0} & \mathbf{C}_\theta^{(n)} \end{pmatrix} \tag{13}$$

$$Q_k = \begin{pmatrix} \mathbf{0} & & \cdots & & \mathbf{0} & \mathbf{0} \\ & \ddots & & & & \\ \vdots & & Q_j^{(i)} & & \vdots & \vdots \\ & & & \ddots & & \\ \mathbf{0} & & \cdots & & \mathbf{0} & \mathbf{0} \\ \mathbf{0} & & \cdots & & \mathbf{0} & \mathbf{0} \end{pmatrix} \tag{14}$$

7.1.3. Expectation-Maximization

After determining the probability distribution for the generative model eq. (11), we estimate the model parameters using the variational approach targeting the lower bound of the cost function, eq. (15). In this equation we create the variational distribution q, which should converge to the posterior distribution of the parameter $p(\boldsymbol{\theta}|\mathbf{y})$.

$$\mathcal{L} = \ln p(\mathbf{y}) = \ln \int d\boldsymbol{\theta}\, p(\mathbf{y}, \boldsymbol{\theta}) = \ln \int d\boldsymbol{\theta}\, q(\boldsymbol{\theta}) p(\mathbf{y}, \boldsymbol{\theta})/q(\boldsymbol{\theta}) \tag{15}$$

Using the Jensen's inequality Jensen (1906) for concave distributions, the eq. (15) becomes

$$\mathcal{L} \geq \int d\boldsymbol{\theta}\, q(\boldsymbol{\theta}) \ln \left[p(\mathbf{y}, \boldsymbol{\theta}) / q(\boldsymbol{\theta}) \right]$$

We define the lower bound of the cost function $\mathcal{L}$ by

$$\mathcal{F} \;=\; \int d\boldsymbol{\theta}\, q(\boldsymbol{\theta}) \ln p(\mathbf{y}, \boldsymbol{\theta}) - \int d\boldsymbol{\theta}\, q(\boldsymbol{\theta}) \ln q(\boldsymbol{\theta})$$

This equation is known as the free energy equation in thermodynamics. The second term is the entropy, S_θ, and has a convenient closed form for a Gaussian distribution: $S_\theta \;=\; -\frac{1}{2} \ln |\mathbf{C}_{\theta|y}| + const$ using the posterior distribution over the parameters. Taking the first term of the lower bound $\mathcal{F}$:

$$\begin{aligned}
\int d\boldsymbol{\theta}\, q(\boldsymbol{\theta}) \ln p(\mathbf{y}, \boldsymbol{\theta}) &= \tfrac{1}{2} E\left[-d \times \ln 2\pi - \ln |\mathbf{C}_\epsilon| - (\bar{\mathbf{y}} - \bar{\mathbf{X}}\boldsymbol{\theta})^T \mathbf{C}_\epsilon^{-1} (\bar{\mathbf{y}} - \bar{\mathbf{X}}\boldsymbol{\theta}) \right]_q \\
&= -\tfrac{1}{2} \ln |\mathbf{C}_\epsilon| - \tfrac{1}{2} E\left[(\mathbf{r} - \bar{\mathbf{X}}(\boldsymbol{\theta} - \boldsymbol{\eta}_{\theta|y}))^T \mathbf{C}_\epsilon^{-1} (\mathbf{r} - \bar{\mathbf{X}}(\boldsymbol{\theta} - \boldsymbol{\eta}_{\theta|y})) \right]_q + const. \\
&= -\tfrac{1}{2} \ln |\mathbf{C}_\epsilon| - \tfrac{1}{2} \mathbf{r}^T \mathbf{C}_\epsilon^{-1} \mathbf{r} - \tfrac{1}{2} E\left[(\bar{\mathbf{X}}(\boldsymbol{\theta} - \boldsymbol{\eta}_{\theta|y}))^T \mathbf{C}_\epsilon^{-1} (\bar{\mathbf{X}}(\boldsymbol{\theta} - \boldsymbol{\eta}_{\theta|y})) \right]_q + const.
\end{aligned} \tag{16}$$

Where $E\left[\cdot\right]_q$ represents the expectation over the distribution q, and $\mathbf{r} \;=\; \bar{\mathbf{y}} - \bar{\mathbf{X}}\boldsymbol{\eta}_{\boldsymbol{\theta}|\boldsymbol{y}}$. In the second level of the system of equations we created appears the posterior momentum $\boldsymbol{\eta}_{\theta|y}$. d is the dimension of the outcome vector. The last term can be simplified into:

$$E\left[(\bar{\mathbf{X}}(\boldsymbol{\theta} - \boldsymbol{\eta}_{\theta|y}))^T \mathbf{C}_\epsilon^{-1} (\bar{\mathbf{X}}(\boldsymbol{\theta} - \boldsymbol{\eta}_{\theta|y})) \right]_q \;=\; E\left[tr\{ \bar{\mathbf{X}}^T \mathbf{C}_\epsilon^{-1} \bar{\mathbf{X}} (\boldsymbol{\theta} - \boldsymbol{\eta}_{\theta|y})) (\boldsymbol{\theta} - \boldsymbol{\eta}_{\theta|y}))^T \} \right]_q \;=\; tr\{ \bar{\mathbf{X}}^T \mathbf{C}_\epsilon^{-1} \bar{\mathbf{X}} \mathbf{C}_{\theta|y} \}$$

The estimation of the trace is not the the sum of the diagonal of an identity matrix. In the expectation-maximization estimation, the terms of the trace are not estimated at the same step. The lower bound can be re-written into:

$$\mathcal{F} \;=\; -\frac{1}{2} \ln |\mathbf{C}_\epsilon| - \frac{1}{2} \mathbf{r}^T \mathbf{C}_\epsilon^{-1} \mathbf{r} - \frac{1}{2} tr\{ \bar{\mathbf{X}}^T \mathbf{C}_\epsilon^{-1} \bar{\mathbf{X}} \mathbf{C}_{\theta|y} \} + \frac{1}{2} \ln |\mathbf{C}_{\theta|y}| + const \tag{17}$$

We used the Expectation-Maximization algorithm Baum et al. (1970); Dempster et al. (1977) to iteratively access the posterior momenta (E-step), maximizing the likelihood (M-step).

E-step. The step maximizes the lower bound, eq. (17), with respect of the variational density q. In other words, we are taking the posterior momenta, eq. (12), and using them in the M-step.

M-step. In this step, we are estimating the extrema of the lower bound, eq. (17), using the parameters from the E-step. The update of the hyper-parameters is performed using the Fisher scoring algorithm. First, we derive the gradient g and the expected second partial derivatives (or Fisher's Information matrix) H:

$$\begin{aligned}
g_k &= \frac{\partial \mathcal{F}}{\partial \lambda_k} &&= -\tfrac{1}{2} e^{\lambda_k} \left(tr(PQ_k) - \bar{\mathbf{y}}^T P^T Q_k P \bar{\mathbf{y}} \right) \\
H_{kl} &= E\left[\frac{\partial^2 \mathcal{F}}{\partial \lambda_k \partial \lambda_l} \right] &&= \tfrac{1}{2} e^{\lambda_k + \lambda_l} tr\left(PQ_k PQ_l \right) \\
& \quad P &&= \mathbf{C}_\epsilon^{-1} - \mathbf{C}_\epsilon^{-1} \bar{\mathbf{X}} \mathbf{C}_{\theta|y} \bar{\mathbf{X}}^T \mathbf{C}_\epsilon^{-1}
\end{aligned} \tag{18}$$

The full algorithm alternates between the E- and M-steps until convergence.

7.2. Cluster volume analysis on cognitively normal subjects

We examined the growth of clusters across the -ACN group by treating each as a participant of interest compared with the rest of the -ACN group (*i.e.* including their first two images the BLME model along with the rest of the -ACN participants, and looking for regions of unexpectedly low volume in subsequent images not included in the model, using the threshold $erf < -0.90$). The emergence of regions of atrophy and growth of clusters of these regions was, on average, smaller than the growth in the +ACN and converter groups, suggesting that large clusters are unlikely to emerge in participants with little chance of developing dementia (Figure 6). It is important to note that the cluster growth in each of these -ACN individuals does not quite represent the same thing as the clusters in the converter and +ACN groups. In the converter and +ACN groups, each participant was included with a control group that was exactly the same for all participants of interest. For the analysis that examined clusters in the -ACN group, the control group was slightly different for each participant, in that the images beyond the first two from the -ACN participant being examined as a participant of interest were excluded from the BLME model. In Figure 6, the panels on the left and right are the cluster sizes over time for the converters and +ACN groups, respectively, and the middle panel shows clusters over time for the -ACN group.

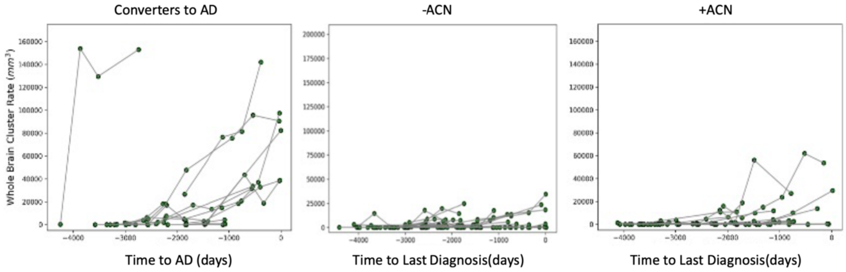

Figure 6: Comparison of the cluster growth between the three cohorts used in the manuscript. We observe the cognitively normal cohort (-ACN, middle plot) shows less cluster growth compared to the two other categories: converters (left plot) and +ACN (right plot).

7.3. Neuropsychological Assessments for Cases 1 and 2

The neuropsychological battery used to assess these cases has been described elsewhere Kramer et al. (2003a) and included the Mini-Mental State Examination (MMSE Folstein et al. (1975)); a copy of the Benson complex figure Kramer et al. (2003a) to assess visuospatial function, backward digit span Wechsler (1997); a 15-item Boston Naming Test Kaplan et al. (1983); phonemic fluency (words beginning with the letter 'D'/minute Kramer et al. (2003a)); semantic fluency (animals/minute Delis et al. (2001); the California Verbal Learning Test, Second Edition (CVLT DELIS (2000)), Standard Form (16 word list) or Short Form (9 word list); and a test of memory for the Benson figure.

7.4. ADNI Converters

The Figure 7 represents the cluster maps over time on the same axial cut for each individual in the Converter group.

7.5. DIAN Consortium Members

Table 6: DIAN Consortium Members.

Name	Institution
Ricardo Allegri	FLENI Institute of Neurological Research*
Randall Bateman	Washington University School of Medicine
Tammie Benziger	Washington University School of Medicine
Sarah Berman	University of Pittsburgh

Jacob Bechara	Neuroscience Research Australia
Courtney Bodge	Brown University-Butler Hospital
Susan Brandon	Washington University School of Medicine
William Brooks	Neuroscience Research Australia
Jill Buck	Indiana University
Virginia Buckles	Washington University School of Medicine
Sochenda Chea	Mayo Clinic Rochester
Jasmeer Chhatwal	Brigham and Women's Hospital-Massachusetts
Patricio Chrem	FLENI Institute of Neurological Research*
Helena Chui	University of Southern California
Jake Cinco	University College London
Carlos Cruchaga	Washington University School of Medicine
Tamara Donohue	Washington University School of Medicine
Jane Douglas	University College London
Noella Edigo	FLENI Institute of Neurological Research*
Nilufer Erekin-Taner	Mayo Clinic Jacksonville
Anne Fagan	Washington University School of Medicine
Marty Farlow	Indiana University
Colleen Fitzpatrick	Brigham and Women's Hospital-Massachusetts
Gigi Flynn	Washington University School of Medicine
Nick Fox	University College London
Erin Franklin	Washington University School of Medicine
Hisako Fuji	Osaka City University
Cortiga Gant	Washington University School of Medicine
Samantha Gardener	Edith Cowan University, Perth, Australia
Bernardino Ghetti	Indiana University
Alison Goate	Icahn School of Medicine at Mount Sinai
Jill Goldman	Columbia University
Brian Gordon	Washington University School of Medicine
Neill Graff-Radford	Moyo Clinic Jacksonville
Julia Gray	Washington University School of Medicine
Alexander Groves	Washington University School of Medicine
Jason Hassenstab	Washington University School of Medicine
Laura Hoechst-Swisher	Washington University School of Medicine
David Holtzman	Washington University School of Medicine
Russ Hornbeck	Washington University School of Medicine
Siri Houeland DiBari	German Center for Neurodegenerative Diseases (DZNE) Munich
Takeshi Ikeuchi	Niigata University
Snezana Ikonomovic	University of Pittsburgh
Clifford Jack	Mayo Clinic Rochester
Gina Jerome	Washington University School of Medicine
Mathias Jucker	German Center for Neurodegenerative Diseases (DZNE) Munich
Celeste Karch	Washington University School of Medicine
Kensaka Kasuga	Niigata University
Takeshi Kawarabayashi	Hirosaki University
Willian Klunk	University of Pittsburgh
Robert Koeppe	University of Michigan
Elke Kuder-Buletta	German Center for Neurodegenerative Diseases (DZNE) Munich
Christophe Laske	German Center for Neurodegenerative Diseases (DZNE) Munich
Jae-Hong Lee	Asan Medical Center
Allan Levey	Emory University School of Medicine
Johannes Levin	German Center for Neurodegenerative Diseases (DZNE) Munich

Ralph Martins	Edith Cowan University, Perth, Australia
Neal Scott Mason	University of Pittsburgh
Colin Masters	University of Melbourne
Denise Maue-Dreyfus	Washington University School of Medicine
Eric McDade	Washington University School of Medicine
Hiroshi Mori	Osaka City University
John C. Morris	Washington University School of Medicine
Akem Nagamatsu	Tokyo University
James Noble	Columbia University
Joanne Norton	Washington University School of Medicine
Richard Perrin	Washington University School of Medicine
Marc Raichle	Washington University School of Medicine
Alan Renton	Icahn School of Medicine at Mount Sinai
John Ringman	University of Southern California
Jee Hoon Roh	Asan Medical Center
Stephen Salloway	Brown University-Butler Hospital
Peter Schofield	Neuroscience Research Australia
Hiroyuki Shimada	Osaka City University
Wendy Sigurdson	Washington University School of Medicine
Hamid Sohrabi	Edith Cowan Univeristy, Perth, Australia
Paige Sparks	Brigham and Women's Hospital-Massachusetts
Kazushi Suzuki	Tokyo University
Kevin Taddei	Edith Cowan Univeristy, Perth, Australia
Peter Wang	Washington University School of Medicine
Chengjie Xiong	Washington University School of Medicine
Xioung Xu	Washington University School of Medicine

* Fundacion para la Lucha contra las Enfermedades Neurologicas de la Infancia

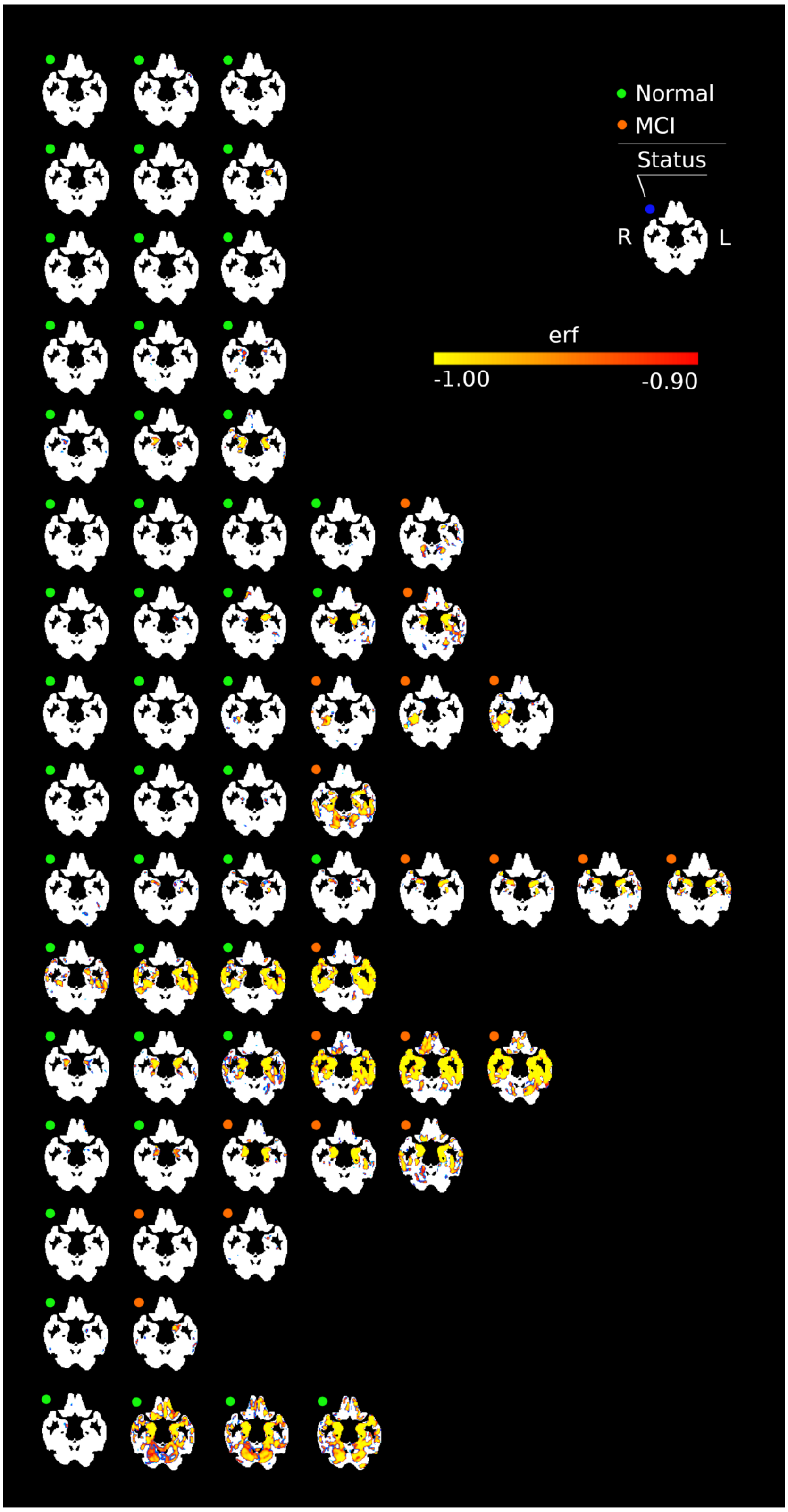

Figure 7: Converter cluster maps over time. Green dots denote images that were acquired at a time when the participant had normal cognition, orange dots denote images collected at the MCI stage.
